# Supplementary material for: Borrelia burgdorferi and Borrelia miyamotoi seroprevalence in California blood donors
Source: PLoS One. 2020 Dec 28;15(12):e0243950. doi: 10.1371/journal.pone.0243950 (PMC7769429; doi:10.1371/journal.pone.0243950)
Supplement: S1 Raw images — (PDF) [file pone.0243950.s001.pdf]

Brummit et al S1_raw_images: Western Blot Image specifications

Raw blot images for Figure 3, Brummitt et al, *Borrelia burgdorferi and Borrelia miyamotoi, seroprevalence in California Blood Donors*

Images in the following pages are the source of the images for Figure 3, containing Run 1, Run 2, Run 3 and the *B. miyamoto*i Glp Q plot.

IgG Western blots were run as described in materials and methods section. Resulting strips were cut in lane order as they were loaded, taped to blank white paper and run through scanner to generate images.

Run numbers and *B. miyamotoi* GlpQ blot names match the names in the figure

Run 1

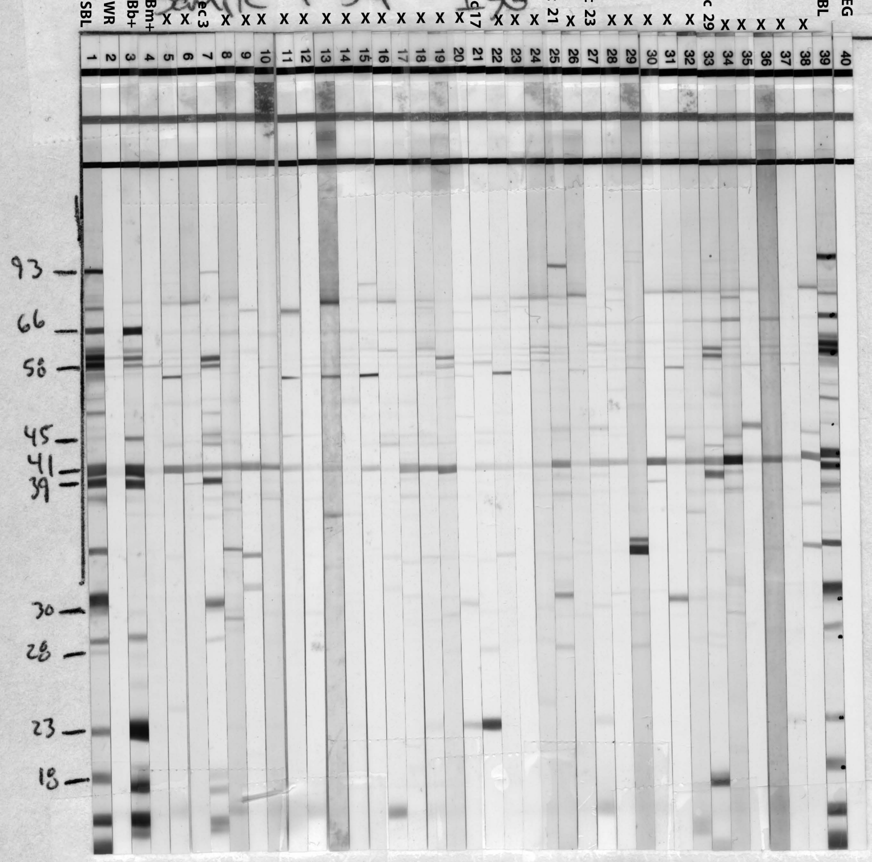
Sample 1-34 IgG
SBL
WR
Bb+
Bm+
Spec 3
Spec 17
Spec 21
Spec 23
Spec 29
SBL
NEG
1 2 3 4 5 6 7 8 9 10 11 12 13 14 15 16 17 18 19 20 21 22 23 24 25 26 27 28 29 30 31 32 33 34 35 36 37 38 39 40
93
66
58
45
41
39
30
28
23
18

Run 2

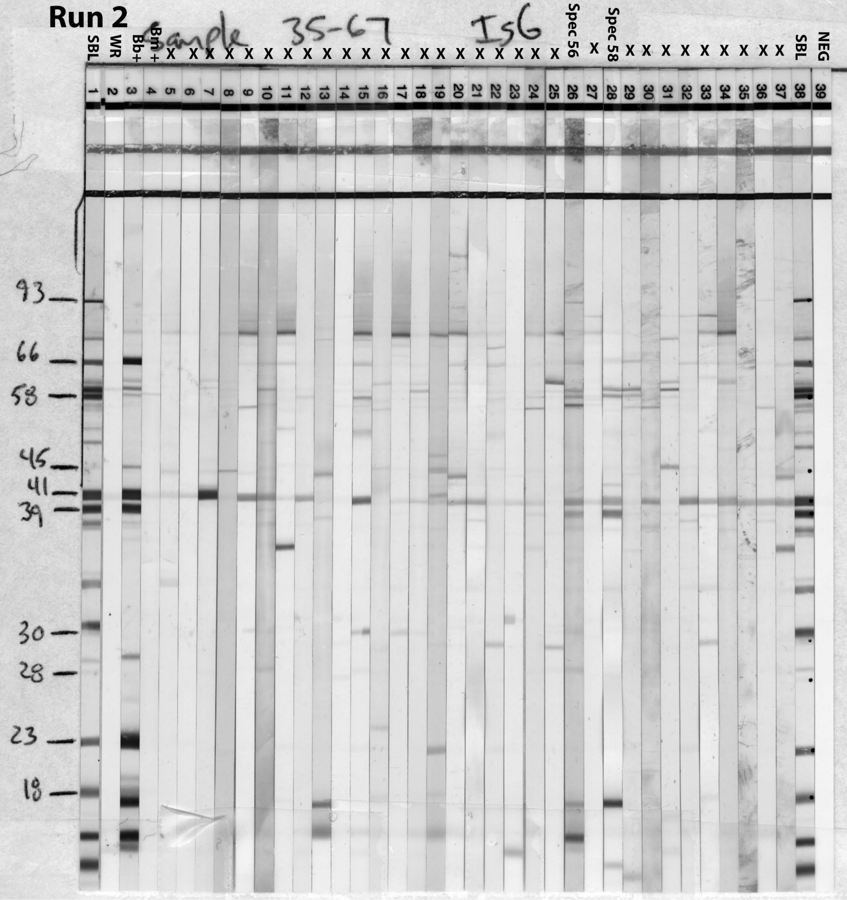
Sample 35-67 IgG
SBL
WR
Bb+
Bm +
Spec 56
Spec 58
SBL
NEG
1 2 3 4 5 6 7 8 9 10 11 12 13 14 15 16 17 18 19 20 21 22 23 24 25 26 27 28 29 30 31 32 33 34 35 36 37 38 39
93
66
58
45
41
39
30
28
23
18

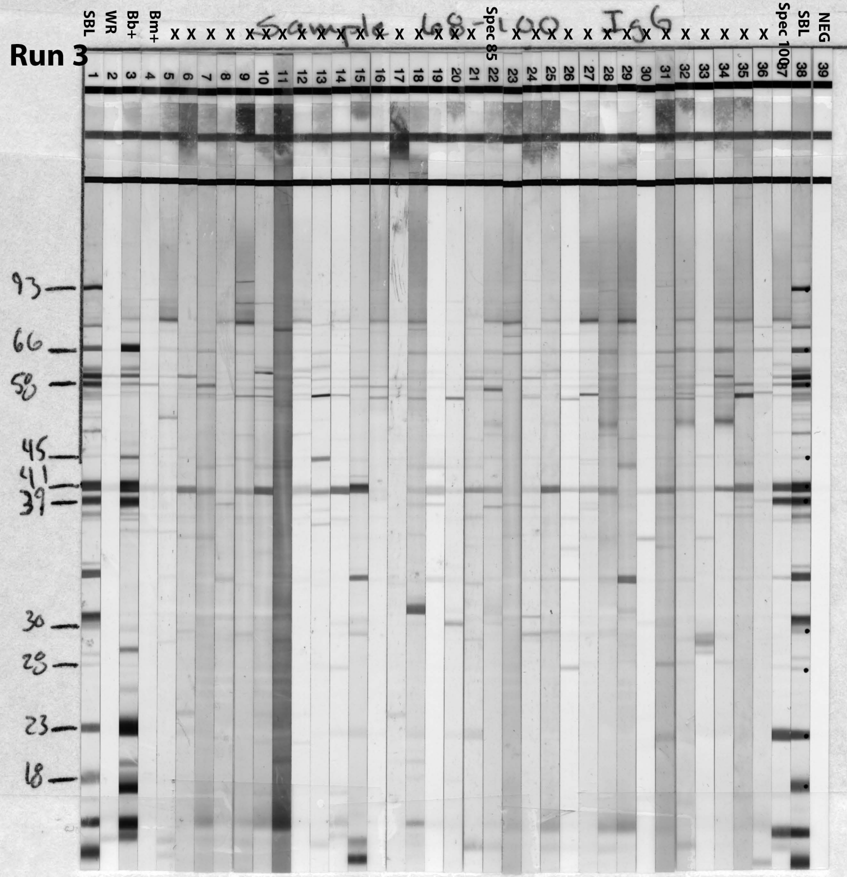
Run 3
Sample 68-100 IgG
SBL
WR
Bb+
Bm+
Spec 85
Spec 100
SBL
NEG
93
66
58
45
41
39
30
23
18

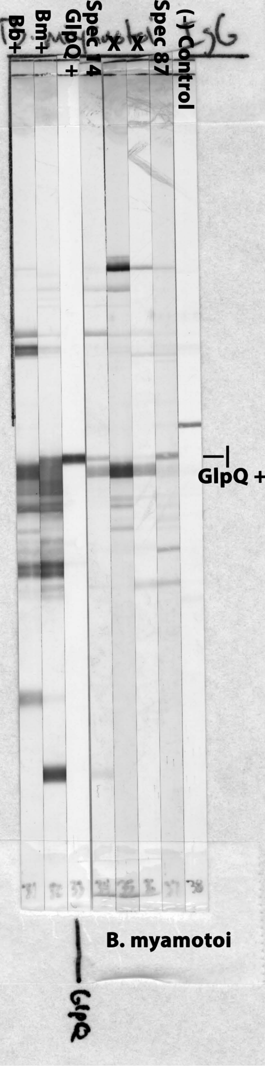

Bb+
Bm+
GlpQ +
Spec 14
X
X
Spec 87
(-) Control
GlpQ +
B. myamotoi
GlpQ
