# Supplementary material for: Borrelia burgdorferi and Borrelia miyamotoi seroprevalence in California blood donors
Source: PLoS One. 2020 Dec 28;15(12):e0243950. doi: 10.1371/journal.pone.0243950 (PMC7769429; doi:10.1371/journal.pone.0243950)
Supplement: Attachment — Submitted filename: PlosOne 2020 Brummitt etal.doc [file pone.0243950.s002.doc]

PONE

Review: *Borrelia burgdorferi* and *Borrelia miyamotoi* Seroprevalence in California Blood Donors.

This study assessed the seroprevalence of B. burgdorferi and B. miyamotoi in 1700 blood donors in California. Donors were selected from counties with reported high prevalence of B. burgdorferi infection as well as those with low prevalence. Of the 1700 donors, 91 were positive (or equivocal) by a standalone C6 ELISA assay for B. burgdorferi. When two tier (STTT or MTTT) assay was applied only 8 of the 91 donors were positive for B. burgdorferi antibodies. Two donors were positive for B. miyamotoi antibodies. No major differences in seroprevalence were observed between high and low prevalence areas. In total 0.47% of patients had serological evidence of B. burgdorferi infection and 0.12% had serological evidence of B. miyamotoi infection. The findings demonstrate low prevalence of B. burgdorferi and B. miyamotoi infection in California.

A key strength of this study is the assessment of seroprevalence for both agents at two independent testing sites (UCD and CDC) and the use of 2 testing methodologies (STTT and MTTT) for B. burgdorferi. The results are largely consistent with previous findings. The major comment is regarding the data presentation in the Results, which could be improved.

1) Figure 1 should include the number of samples that came from each county as well as the percentage. Figure Legends should be provided.

2) The 2nd paragraph of the Results (C6 ELISA Screen) provides information on how the 91 patients were selected for downstream analyses, but this information is not presented anywhere. My suggestion is to present this in a form of a Table or a flow chart since all subsequent analyses in the manuscript stem from this selection/screen.

3) In the second paragraph of the C6 ELISA Screen section, the authors discuss the distribution of Hispanics and non-Hispanics among low and high prevalence areas in California. They make a point that individuals with positive / equivocal result were usually non-Hispanic however one would assume this based on the lower prevalence of Hispanics in this cohort. The seroprevalence to C6 peptide should be assessed directly in Hispanics vs non-Hispanics taking into account the sample size for each.

4) The authors selected randomly 9 negative samples by standalone C6 ELISA, two thirds of which were female. What is the rationale for selecting 9 samples (vs more) and why were they not gender matched with the C6 seropositive populations?

5) Table 2 should include a row that lists the C6 findings for all 1700 patients. It should also include data for all 941 samples from high endemic area, as well as for 759 patients from low enedemic area; followed by the data for each county in that area. The data need to be clearly labeled (what are the data in parenthesis? Do the data represent positive results?). It would help to present the number of positives in addition to percentages.

6) The paragraph on B. miyamotoi GlpQ seroreactivity discusses seroprevalence in high vs low endemic regions for B. burgdorferi. This discussion is based on only two positive samples, and it is difficult to conclude much in the difference in prevalence between the two regions, except that the seroprevalence is relatively low. One could also postulate that the infection could have been acquired elsewhere and not in the counties/state where the samples were obtained.

7) A similar comment holds true for the paragraph on B. burgdorferi IgG western blot. The authors provide percentages and CI for seroprevalence in Marin county, but only one donor was seropositive there. With such small numbers it is difficult to assess the significance of seroprevalence and one should be careful to not overinterpret the data. For example, is it fair to state that San Luis Obispo county had the highest prevalence in the low endemic area since it had the only positive case (out of 759) in this region?

8) Line 369 in the Discussion: is it low specificity or high sensitivity of C6 ELISA that would over-estimate true exposure?
